# Upper Lip Basal Cell Carcinoma Reconstruction Based on Anatomical Characteristics Using Skin-Mucosa Double Opposing V-Y Advancement Flap

**Published:** 2011-05-13

**Authors:** Dinh T. Nguyen, Koichiro Oki, Hiko Hyakusoku, Rei Ogawa

**Affiliations:** ^a^Department of Plastic, Reconstructive and Aesthetic Surgery, Nippon Medical School Hospital, Tokyo, Japan; Dr Nguyen is in private practice at Boston, MA.

## Abstract

**Background:** Upper lip defects less than one-fourth of total upper lip length are typically closed directly, but larger defects require reconstruction. Established techniques, such as the Abbe/Estlander flap, often require multiple procedures. This report discusses a single-step method, involving a skin-mucosa double opposing V-Y advancement flap, which was utilized to reconstruct the upper lip after basal cell carcinoma extirpation. **Methods:** The patient is a 54-year-old woman who presented with a 2.5-cm basal cell carcinoma in the right upper lip. Two parallel V-Y flaps were designed, on “parallel” planes of the skin and mucosa, and were advanced following wedge resection of tumor and portion of the adjacent orbicularis oris muscle. Skin/mucosa flap edges were brought to create a new skin-vermilion border, which was then sutured onto existing skin-vermilion border edges. **Results:** The patient was evaluated 6 months after the operation, and examination showed well-healed flaps with excellent aesthetic and functional results. There was a thin, marginal scar, where reconstructed vermilion (medial) edge was sutured to native vermilion. **Conclusions:** It was considered that this method could reconstruct anatomical features and function of the lip precisely. It seems that within certain limits (probably between one-fourth and one-third of total upper lip length), skin-mucosa double opposing V-Y advancement flaps appear to be the preferred method for upper lip reconstruction.

The upper and lower lips are key features that define our physical appearance. Moreover, they play a critical role in facial expression, speech formulation, and food consumption. Therefore, proper restoration of lip form and function postinjury is of paramount importance.

Defects to the lips are typically cause by either trauma or neoplasm. Smaller defects to the lips can be closed directly, but larger defects require reconstruction. Established methods of reconstruction yield good results, but often involve multiple steps, which can be cumbersome in certain populations—particularly pediatric patients. Hence, efforts have been made to improve and simplify reconstructive techniques.^1^^-^4 One technique is presented in this report and involves reconstruction of an upper lip defect (from basal cell carcinoma [BCC]) with parallel skin-mucosa double V-Y opposing advancement flap.

## CASE REPORT

The patient is a 54-year-old woman, who presented with a 2.5-cm dome-shaped tumor in the right upper lip. Associated findings include black pigmentation with “pearly” border region—consistent with BCC. The patient reports that the tumor started as a small lesion about 2 years earlier. Preliminary clinical staging of the tumor was stage I (T1N0M0).

**Figure 1:**
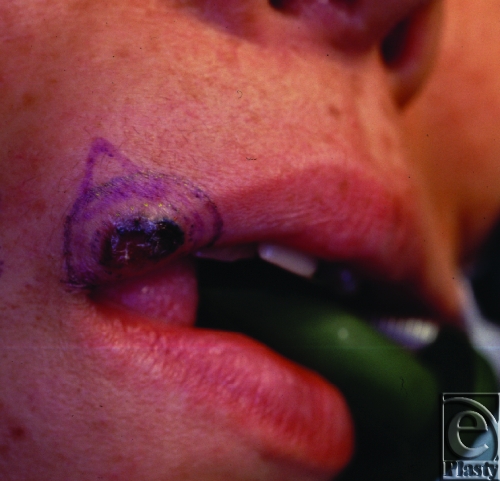
Preoperative view: Upper lip tumor with characteristics of basal cell carcinoma.

**Figure 2:**
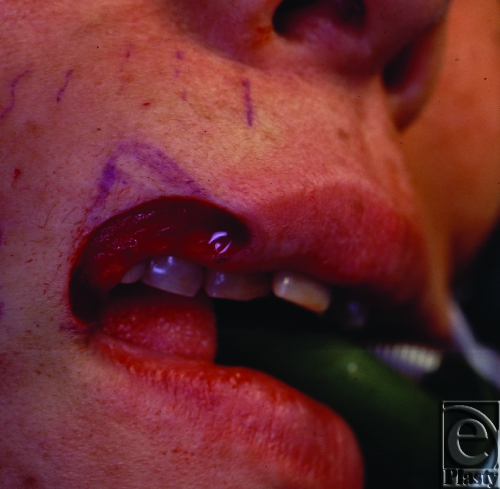
Intraoperative view: Tumor extirpation with 5 mm margin of normal skin and orbicularis oris muscle.

**Figure 3:**
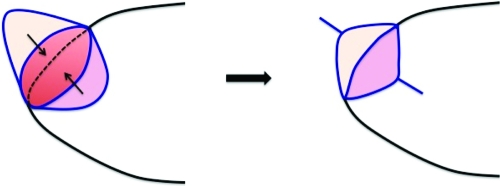
Two parallel V-Y flaps: Two parallel V-Y flaps were designed, on “parallel” planes of the skin and mucosa, and were advanced following wedge resection of tumor and portion of the adjacent orbicularis oris muscle. Skin/mucosa flap edges were brought to create a new skin-vermilion border, which was then sutured onto existing skin-vermilion border edges.

**Figure 4:**
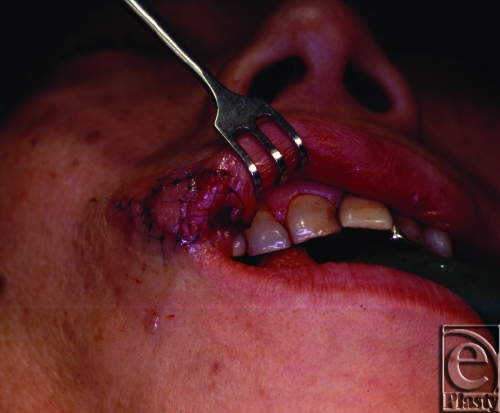
Immediate postoperative view: Double opposing V-Y flap, harvested from skin and mucosa, were sutured together to create a white lip-vermilion border.

A decision was subsequently made to extirpate the tumor, with a 5-mm normal skin margin, followed by immediate reconstruction (Fig 1). Two parallel V-Y flaps were designed (Fig 2), on “parallel” planes of the skin and mucosa and were advanced following wedge resection of the tumor and portion of the adjacent orbicularis oris muscle by scalpel (Fig 3). Skin/mucosa flap edges were brought to create a new skin-vermilion border, which was then sutured onto existing skin-vermilion border edges (Fig 4). Respective skin and mucosa/vermilion edges were brought together, with subcutaneous tissue strategically manipulated to create bulkiness. The patient tolerated to procedure without any complications. Postoperative results were satisfactory.

**Figure 5:**
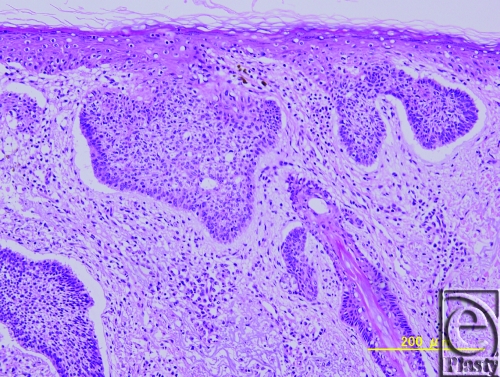
Pathological findings: Histopathological examination revealed typical basal cell carcinoma. Also visualized is connective tissue stroma (parallel bundels of young fibroblasts) surrounding tumor islands.

Basal cell carcinoma was later confirmed by histopathologic findings (Fig 5). Microscopic examination also revealed that the connective tissue stroma, surrounding tumor islands, was arranged in parallel bundles with young fibroblasts immediately adjacent to the tumor itself. Moreover, retraction of stroma from tumor islands was also visualized. Horizontal and deep margins were intact.

**Figure 6:**
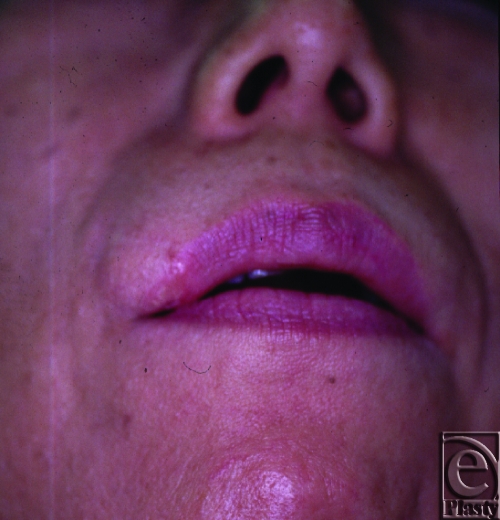
Six-month postoperative view: Vermillion border is clearly reconstructed and the lip is symmetrical. A small scar, from suturing reconstructed vermilion edge to native vermilion, is also visualized.

The patient was evaluated 6 months after the operation, and examination showed well-healed flaps with excellent aesthetic and functional results (Fig 6). There was a thin, marginal scar where reconstructed vermilion (medial) edge was sutured to native vermilion. Reconstructed vermilion texture closely resembled that of native vermilion, while lip symmetry and coloration was maintained. It is worth mentioning that no tumor recurrence was noted during the 3-year follow-up visit with the surgical oncology service. Lip appearance, sensation, and movement were essentially normal.

## DISCUSSION

As aforementioned, in addition to being a main feature of physical appearance, the lips also play a key role in facial expression, speech formulation, and food consumption. Anatomically, cross-sectional layers of the (upper and lower) lips are composed of skin/vermilion, subcutaneous tissue, muscle, submucosa, and mucosa.^5^^-^7

The vermilion is a unique structure associated with the lips and has properties distinct from the oral mucosa, including color, texture, and light reflectivity.^6^ Its red appearance can be attributed to a rich supply of capillaries that lies beneath a layer of nonkeratinized, specialized squamous epithelium (lacking hair and sebaceous glands).^7^

Within the oral cavity, the internal lips are lined by mucosa (nonkeratinized epithelium, rich in minor salivary glands), which smoothly transitions into vermilion a few millimeters behind the natural mouth closure.^6^ The vermilion may be subdivided into wet and dry zones, whereby the border is termed wet-dry line. The vermilion extends to meet a musculocutaneous ridge (formed by the orbicularis oris muscle complex) known as the white role. Skin connected to the white role extends to the nasal base, nasolabial folds, and labiomental crease. A significant amount of subcutaneous tissue lies deep to the skin, making up the bulk of lip thickness.

Originating just lateral to the commissure (where the upper and lower lips meet), at the modiolus (crossroad of several facial muscles), the most important muscle(s) of the lips is a pair of orbicularis oris, sometimes referred to as orbicularis oris complex.^6^ Their fibers join at the midline of the lower lip (in a raphe). In the upper lip, fibers cross the midline and inserts into the opposite philtral column. Although the majority of fibers are horizontally aligned, the orbicularis have sphincteric characteristics and is innervated by the buccal branch of the facial nerve (CN VII). In cross-lip flaps, the orbicularis oris complex is cut and rearranged, and thus, muscle dysfunction can occur. In contrast, such complication is avoided with direct closure (after advancement), as is the case in the presented method.

Several other facial muscles are associated with lip movement. Lip elevators are levator labii superioris, levator anguli oris, and zygomaticus major/minor.^7^ Lip depressors are depressor labii inferioris and depressor anguli oris. The mentalis elevates and protrudes the lower lip and the risorius retracts the angle of the mouth. These muscles are innervated by branches of the facial nerve.

Sensory innervation to the lips is provided by the maxillary division of the trigeminal nerve (V_2_) and the submandibular division of the trigeminal nerve (V_3_).^6^ The infraorbital nerve, a branch of the maxillary division, innervates the upper lips. The mental nerve, a terminal branch of the inferior alveolar nerve (a branch of the submandibular division), innervates the lower lips. Unlike cross-lip flaps, which render transposed lip tissue nonsensate, the presented method allows for the preservation of nerves (as well as perforator vessels), which in turn, allows for rapid recovery of lip sensation.^2^ The very act of eating and drinking relies on sensory restoration.

The lips are supplied by branches of the facial artery.^7^ The superior and inferior labial arteries provide primary arterial supply to the upper and lower lips, respectively. Originating deep to the depressor anguli oris and orbicularis oris, they course subjacent along the contours of the orbicularis oris muscle complex, forming a complete loop, while sending perforators (through the orbicularis oris itself) to supply the overlying skin/vermilion.^6^ In cross section, the labial artery is found about the region of the wet-dry line. Venous drainage does not generally follow arterial supply, but networks merge at nearby major/named arteries to form larger veins.

As mentioned earlier, lip defects are typically caused by either trauma or tumors. Trauma usually occurs with young adults and tumors can be either acquired or congenital.^6^ Acquired tumors are typically BCC in the upper lip and squamous cell carcinoma in the lower lip (due to increased sun exposure). Melanoma also occurs, but with less frequency.

Regardless of defect etiology, aesthetic and functional significance of the lips requires appropriate reconstruction. Depending on defect size and location, several techniques can be utilized to ensure proper restoration of form and function. Improper reconstruction not only causes lip deformity and dysfunction, but it may also lead to psychoemotional anguish.

In closing lip defects, several factors must be considered, including defect size and remaining vermilion.^6^ In general, defects larger than 40% of both (upper and lower) lips combined or larger than 80% of either lip requires reconstruction with non-lip tissue to avoid microstomia.^5^^-^6

Upper lip defects that are less than one-fourth of the upper lip length can be closed directly.^5^^-^6 Primary closure of larger defects often leads to asymmetry and/or “whistling deformity.” This dilemma can be rectified with local cross-lip flap, particularly the Abbe flap^8^ or Estlander flap.^9^^-^10

If the defect is less than one third, reconstruction with a cross-lip flap can be avoided.^5^^-^6 In such cases, a one-step V-Y advancement of local tissue can be employed to reconstruct the upper lip. Lower lip reconstruction using this method has been successful (aesthetically and functionally) in a series of patients with lower lip defects from cancer (confined between skin and vermilion).^4^ Compared to the Abbe/Estlander flap, V-Y advancement is technically less challenging yet yield similar outcomes.^1^^-^3 Because of the vicinity of harvested skin, reconstructed lip skin color/texture (with few exceptions) resembles that of the adjacent native skin in almost everyway. Moreover, reconstructed vermilion resembles that of the adjacent native vermilion while maintaining overall lip symmetry. Like cross-lip flaps, displacement of the commissure is avoided.

To conclude, the success of the skin-mucosa double opposing V-Y advancement flap relies on postprocedural sensory and motor function capacities as well as aesthetic lip appeal. However, extreme care must be taken to ensure that complete tumor excision with adequate margins has been performed, especially if the method applied to the lower lips (where prevalence of more aggressive squamous cell carcinoma is relatively higher). While it is sensible to presume that the technique is more likely to yield long-term clinical resolution in excisions that causes less tissue disruption and BCC subtypes with histopathologic findings that are more localized; however, the current small data set precludes analysis for validation.

Although the number of operations performed is still relatively small, surgical outcomes from skin-mucosa double opposing V-Y advancement flap for reconstruction of upper lip defects have been good and are comparable to cross-lip flaps. Therefore, it seems that within certain limits (probably between one fourth and one third of total upper lip length), skin-mucosa double opposing V-Y advancement flap appears to be the preferred method for upper lip reconstruction.
